# Hydroxylation at Multiple Positions Initiated the Biodegradation of Indeno[1,2,3-cd]Pyrene in *Rhodococcus aetherivorans* IcdP1

**DOI:** 10.3389/fmicb.2020.568381

**Published:** 2020-09-22

**Authors:** Li-Li Miao, Jie Qu, Zhi-Pei Liu

**Affiliations:** ^1^State Key Laboratory of Microbial Resources, Institute of Microbiology, Chinese Academy of Sciences, Beijing, China; ^2^Shandong Key Laboratory of Biophysics, Shandong Engineering Laboratory of Porcine Health Big Data and Intelligent Monitoring, Institute of Biophysics, Dezhou University, Dezhou, China

**Keywords:** indeno[1,2,3-cd]pyrene, biodegradation, *Rhodococcus aetherivorans* IcdP1, aromatic ring-hydroxylating oxygenase, degradation pathways, ring hydroxylation

## Abstract

Nowadays, contamination by polycyclic aromatic hydrocarbons (PAHs) has become a serious problem all over the world; in particular, high-molecular-weight PAHs (HWM PAHs, four to seven rings) are more harmful to human health and environment due to their more complex structure and metabolic pathway. Biodegradation of PAHs with six or more rings, such as indeno[1,2,3-cd]pyrene (IcdP), was rarely described. An IcdP-degrading strain, *Rhodococcus aetherivorans* IcdP1, was isolated from HWM PAH-contaminated soil. It could grow on and efficiently degrade various HWM PAHs, such as IcdP, benzo[a]pyrene, and benzo[j]fluoranthene. It showed highest degrading ability toward IcdP (> 70% within 10 days). The IcdP degradation was initiated by ring hydroxylation with multiple pathways, including the hydroxylation at the 1,2 and 7,8 positions, according to the relevant metabolites detected, e.g., cyclopenta[cd]pyrene-3,4-dicarboxylic acid and 2,3-dimethoxy-2,3-dihydrofluoranthene. The transcriptional patterns of the genes encoding ring-hydroxylating oxygenases (RHOs) and cytochrome P450 monooxygenases (CYP450s) under the induction of IcdP, pyrene, and benzo[b]fluoranthene (BbF) were compared to determine the key initial RHOs in the conversion of IcdP. The expression of genes encoding RHOs 1892–1894, 1917–1920, and 4740–4741 was induced strictly by IcdP, and the amino acid sequences of these proteins showed very low identities with their homologs. These results suggested that IcdP was degraded through a dioxygenation-initiated metabolism pattern, and RHOs 1892–1894, 1917–1920, and 4740–4741 responded to the initial ring cleavage of IcdP through 1,2-dihydrodiol or 7,8-dihydrodiol. The studies would contribute to the understanding of the molecular mechanism of initial degradation of IcdP.

## Introduction

Polycyclic aromatic hydrocarbons (PAHs), composed of two or more aromatic rings in a clustered or linear pattern, are ubiquitous, hazardous, and persistent organic pollutants widespread in nature. These highly degradation-resistant contaminants commonly exist at sites associated with petroleum, gas production, and wood processing industries (Kuppusamy et al., 2016a). High-molecular-weight PAHs (HWM PAHs, four to seven rings), such as indeno[1,2,3-cd]pyrene (six rings), are more toxic due to their more complex structure and metabolic pathway and labeled hazardous by US EPA in 2008 (Keith and Telliard, 1979; Burchiel and Luster, 2001; Samanta et al., 2002; Kuppusamy et al., 2016b; Wong et al., 2018; Wirnkor et al., 2019). Morillo et al. determined the degree of contamination with PAHs in samples of soil from three European cities. The concentration of indeno[1,2,3-cd] pyrene was in the range 0.158–2.827 mg/kg (Morillo et al., 2007). However, its content was much higher in related industry areas, especially coking plant (see below). Compared to chemical oxidation, physical adsorption, and photooxidation, bioremediation technology has gained increasing attention and is considered to be a cost-effective and eco-friendly method for the removal of PAHs (Haritash and Kaushik, 2009).

PAH degradation pathway was composed of a ring cleavage process (RCP), a side chain process (SCP), and a central aromatic process (CAP) (Kweon et al., 2007, 2011). The activation of thermodynamically stable benzene rings and ring cleavage reaction of the corresponding dihydroxylated intermediates occurred in the RCP, side chain removal to produce biological metabolic precursors occurred in the SCP, and CAP connected the metabolic of protocatechuate to the TCA cycle. The hydroxylation process of the aromatic rings was one of the common initial degradation steps and mainly controlled the rates of PAH degradation; this step catalyzed dioxygenases [Rieske non-heme ring-hydroxylating oxygenase (RHO)] or cytochrome P450 monooxygenases (CYP450s) (Luo et al., 2016). Considerable attention has been devoted in recent years to the identification and annotation of RHOs or CYP450s responsible for ring hydroxylation of HMW PAHs (Kweon et al., 2014). A significantly high number of gene copies encoding RHOs (21 copies) and CYPs (50 copies) had been identified in the genome of *M. vanbaalenii* PYR-1. Six RHOs and 24 CYP genes related to PAH degradation were identified in *Rhodococcus* sp. P14 genome. The redundancy of genes that also included the genes encoding other than oxygenases was thought to contribute to the versatile PAH degradation capacity of *M. vanbaalenii* PYR-1 (Kim et al., 2007, 2008, Peng et al., 2020). Several RHOs for PAH degradation were expressed and characterized in *E. coli* (Zeng et al., 2017).

The aromatic ring cleavage by dioxygenases (RHO) was predominant in prokaryotic HMW PAH biodegradation. The RHOs for the degradation of HMW PAHs such as pyrene and fluoranthene showed high substrate specificity, e.g., the RHO *nid*AB was induced by pyrene and catalyzed its degradation; however, the *nid*A3B3 was induced by fluoranthene but not pyrene in *M. vanbaalenii* PYR-1 (Khan et al., 2001). The *Pdo*1 and *Pdo*2 catalyzed the initial degradation of benz[a]anthracene and fluoranthene, respectively, in *Mycobacterium* sp. strain 6PPYR1 (Krivobok et al., 2003); *Phd*AB catalyzed pyrene transformation in *Mycobacterium* sp. SNP11 (Pagnout et al., 2007).

To date, only few bacterial strains were reported to possess degradation capability toward IcdP; they were isolated from a PAH-contaminated area and used in the bioaugmentation process of PAH-contaminated environments (Huang et al., 2016; Kong et al., 2018). Temitavo et al. reported IcdP degradation potentials of several hydrocarbon-degrading bacteria, such as *Campylabacter hominis*, *Bacillus cereus*, etc.; like most PAH-degrading strains, these strains preferred LMW PAH, such as naphthalene (Temitayo et al., 2019). Aerobic heterotrophic bacteria and Cyanobacteria showed very low degradation efficiency for < 0.1 mg/L IcdP (Tersagh et al., 2018). Researches about degradation of indeno[1,2,3-cd]pyrene (six fused rings) are still extremely rare, especially ring hydroxylation, the first step of IcdP catabolism.

In our lab, an IcdP-degrading strain, *Rhodococcus aetherivorans* IcdP1, was isolated from the HMW PAH-contaminated soil of the abandoned Beijing Coking Plant, which had been continuously poisoned by coking chemicals for more than 50 years. A high content of PAHs (297.29 ± 49.5 mg/kg), especially HMW PAHs (156.93 ± 18.2, IcdP > 38 mg/kg), were present (Xu et al., 2014), indicating heavy pollution by these compounds according to US EPA Guidelines (US EPA, 1992). Thus, in this study, IcdP was chosen as targeted PAHs because it was the most abundant and toxic HMW PAHs in contaminated soil. Strain IcdP1 exhibited strong degrading ability toward several HMW PAHs, especially IcdP, and might show great potential in the remediation of HMW PAH-contaminated environments. The present study mainly focused on its IcdP degradation characterizations, identification of the intermediates, as well as determining the key aromatic ring-hydroxylating dioxygenases (RHOs) or CYP450s in the conversion of IcdP or other PAHs to its HMW or LMW PAH metabolites by means of comparing transcriptome. The results might greatly improve our understanding of the molecular mechanism of initial ring hydroxylation of indeno[1,2,3-cd]pyrene degradation and other PAHs and also provide clues for its application in the bioremediation of PAH-contaminated environments.

## Experimental Section

### Strains and Culture Conditions

Strain IcdP1 was isolated from the contaminated soil of the abandoned Beijing Coking Plant in China (Qu et al., 2015). It could grow and efficiently degrade numerous HMW PAHs, such as benzo[a]pyrene and indeno[1,2,3-cd] pyrene (Qu et al., 2015). The complete genome sequence of *Rhodococcus* sp. strain IcdP1 was deposited in GenBank under accession no. CP011341.

Luria-Bertani (LB) broth (g/L): tryptone 10, yeast extract 5, and NaCl 10. Mineral salt medium (MSM, g/L): NH_4_NO_3_ 1.0. MgSO_4_⋅7H_2_O 0.5, KH_2_PO_4_ 0.5, NaCl 0.5, and K_2_HPO_4_ 1.5, pH 7.0. Seed medium was LB and degradation medium was MSM with 10 mg/L different PAHs as a sole carbon source. All experiments were conducted in shaking flask culture, with a fixed liquid volume of 10 ml in each 100 ml flask in triplicate.

Strain IcdP1 was stored at −80°C and transferred to fresh slants and cultivated for 48 h at 30°C. Loopfuls of lawn were inoculated to LB medium and incubated at 30°C for 24 h. Five percent of pre-incubation broth was further inoculated for another 24 h to produce the starter culture. The overall production period of MSM medium culture was 25 days on a rotary shaker at 180 rpm, 30°C with 10 mg/L HWM PAHs as the sole carbon source. For the mRNA extraction, IcdP1 was inoculated to MSM medium with glucose (5 g/L), 10 mg/L HMW PAHs were induced at 48 h, and then the samples were taken at 12 h intervals. The solvent acetone was used as control. All experiments were performed in triplicate or quadruplicate. Cell concentration was estimated by optical density at 600 nm.

### Chemicals and Reagents

Fluranthene (FLA), pyrene (PYR), benz[a]anthracene (BaA), chrysene (CHR), benzo[b]fluoranthene (BbF), benzo[j] fluoranthene (BjF), benzo[a]pyrene (BaP), dibenz[a,c]anthracene (DacA), dibenz[a,h]anthracene, indeno[1,2,3-cd]pyrene, and other PAHs were purchased from Sigma-Aldrich (United States). Bacterial RNA isolation kit, random primers, ribonuclease inhibitors, dNTP mixture, recombinant DNase I, and SYBR Premix Ex Taq were purchased from Takara Biotechnology Co., Ltd. (Dalian, China).

### Degradation of HMW PAHs by Strain IcdP1

Cultures of strain IcdP1 grown on each HWM PAH (FLA, PYR, BaA, CHR, BbF, BjF, BaP, DacA, DahA, and IcdP) were prepared as mentioned above for 5 days, subjected to sonic extraction with the same volume of dichloromethane for 20 min, and rested for 1 h. The organic phase was transferred to another tube. This process was repeated three times. After drying by centrifugal concentration, the organic phase was dissolved in acetonitrile. The residual HMW PAHs were analyzed quantitatively by high-performance liquid chromatography (HPLC, Agilent 1200, United States) on a C18 column (4.6 × 150 mm × 5 μm, Eclipse XDB-C18) under the following conditions: temperature 40°C, flow rate 1.0 ml/min, and wavelength 245 nm. HMW PAHs were identified based on retention time in comparison with their standards (Sigma) in acetonitrile. Mobile phase consisted of acetonitrile 80% and water 20%. All the quantitative analyses were conducted in triplicate.

### Detection and Identification of Metabolites of IcdP Degradation by Gas Chromatography–Mass Spectrometry

Strain IcdP1 was cultured in LB at 30°C for 24 h; after centrifugation, precipitate was washed three times with 0.2 M PBS (pH 7.0). Then, the precipitate was divided into two parts, one was cultured in MSM medium with 10 mg/L IcdP. The other part was cultured in MSM medium and used as negative control. Cell pellets and supernatant from 1 ml of broth were prepared and collected by centrifugation (8000 *g* for 10 min). Then, cell pellets were subjected to sonic extraction and broken in 1 ml of isopropanol, and then after centrifugation (13,000 *g* for 10 min), the isopropanol phase was collected and mixed to the supernatant of broth. The mixtures were diluted to 20 ml by water and solid phase extracted. Then, 20 ml of the mixture was subjected to a packed solid phase extraction column (300 mg Chromabond C18, German) with a flow rate of 1.5 ml/min, followed by 10 ml of water, 10 ml of acetonitrile/water (10:90, v/v), 2.5 ml of acetonitrile/water (20:80, v/v), and 1 ml of acetonitrile/water (30:70, v/v) to remove the impurities. Last, the metabolites were eluted by 3 ml of acetonitrile/water (55:45, v/v) and 2.5 ml of acetonitrile. All the metabolite samples were freeze-dried and resolved in 200 μl of acetone followed by adding 100 μl of derivatization reagent BSTFA + TMCS (99:1) for 1 h at 65°C. The extracts were analyzed by GC-MS (7890A-5975C, Agilent, United States) with an HP-5MS column (30 m × 0.25 mm × 0.25 μm; Agilent). Helium was used as the carrier gas; its flow rate was 1 ml/min. Split stream sampling was applied at a ratio of 1:5. The injector temperature was 280°C. The column temperature program was set as follows: 2 min at 70°C, first ramp 5°C/min to 150°C, and kept for 3 min, and then linearly increased to 290°C at a rate of 10°C/min and 20 min at 290°C.

The mass spectrometer was operated in selective ion monitoring mode with ion electron impact ionization at 70 eV and ion source at 200°C. The scan mode data were analyzed by Enhanced ChemStation software program (Agilent), comparing it with the mass spectra of reference compounds from NIST-05 and WILEY 8-Mass spectral library.

### Total RNA Purification and Reverse Transcription

Cells in 2 ml of broth were harvested by centrifugation (12,000 rpm, 1 min), frozen immediately in liquid nitrogen, and stored at −80°C until processing. RNA extraction was performed using Bacterial RNA isolation kit according to the manufacturer’s instructions. Total RNA concentration was determined by a NanoDrop 2000c UV-vis spectrophotometer (Thermo Fisher Scientific, United States), and aliquots of extracts were subjected to agarose gel electrophoresis to check RNA integrity. To degrade trace amounts of genomic DNA in RNA preparations, RNA was treated with DNase I. DNase I (1 μl; 5 U/ml) was added to a reaction mixture containing 5 μg of RNA, 4.4 μl of 25 mM MgCl_2_, 2 μl of 10 × Buffer (Takara Japan), and DEPC-treated water to give a total volume of 20 μl. The reaction mixture was incubated for 30 min at 37°C, followed by heat denaturation at 65°C for 15 min. Reverse transcription reaction was performed, after heating the RNA sample to 70°C, in a final volume of 25 μl containing 2 μg of total RNA, 1 μl of random primer, 0.5 mM dNTPs, and 200 U M-MLV reverse transcriptase H minus (Promega). The reaction mixture was incubated for 60 min at 42°C and then heated to 65°C for 15 min.

### Real-Time PCR

Real-time polymerase chain reaction (RT-PCR) analyses were performed using cDNA samples as a template, with an ABI 7900HT apparatus (Applied Biosystems, Norwalk, CT). The analyzed genes and their corresponding primer sets are listed in Table 1. Dissociation curves were constructed to test amplification validity. The SYBR^®^ Green I-based qPCR cycling reaction system and protocols employed were those recommended by the manufacturer, briefly: initial denaturation at 95°C for 3 min and then 40 cycles of 95°C for 10 s, 55°C for 20 s, and 72°C for 10 s (Roche). *RecA*, *rpho*, etc., were used as reference genes. Relative gene expressions with or without the induction of HWM PAHs were calculated by the 2^–Δ^ ^Δ^ *^*CT*^* (cycle threshold) method. Each RT-PCR analysis was run in triplicate or quadruplicate to test consistency.

**TABLE 1:** Primers used for RT-PCR.

Gene*	Primer (5′–3′)	
	Forward	Reverse
*recA*	ATCGGTGTGATGTTCGGCT	CACCTTGTTCTTGACGACCT
*rpoB*	CTCACCATCAAGTCGGAC	TCGTTGCGGGAAAGGTT
*1917*	ATCCGCCGAGTTCTACCAGT	AGTCTGCCGTTCCATCCGTA
*1919*	AACCACCGCTACCACGAAT	CACGGACATTGGTCAGCA
*1920*	TCATCCAGTGAGAACACGC	GCATTCCACGACGCAGTT
*1921*	GTGAAGGGCTATCTGCT	GTTGGACAGGGTGAAGA
*1892*	ATGAGTGTTTCCCGACTCGG	GCGGGTCTCA GGGTCGCCGG
*1894*	GCTTCAACAACAGCGAC	ATGTCGCACAGCACTTC
*4526*	CGACGAAACCTATTACACG	TAGGACTTCGGGCTGAA
*818*	AAGTCGTCCATCCGCAGGTA	GTCCGGCAACACCCTCGA
*865*	AGGTGATGATGCTCTACGG	TGAACGACGACTGCAGACG
*4741*	ACGGACGACGCACATTA	GATTTCCTCCACGGTGAACC
*4740*	ACGACGACAGAAGAACCCA	GTGCGTAGGACCAGTTGTG
*1150*	AAGGGTGAGGCGGAGTTCAT	CGATGAGCAGGAGGAAGAA
*4894*	CGCACCTGGTCAACTATCA	AGATGGCGATGCTGTTCT

*Also see these genes in Figure 4.

## Results

### Degradation of HMW PAHs by Strain IcdP1

**FIGURE 1:**
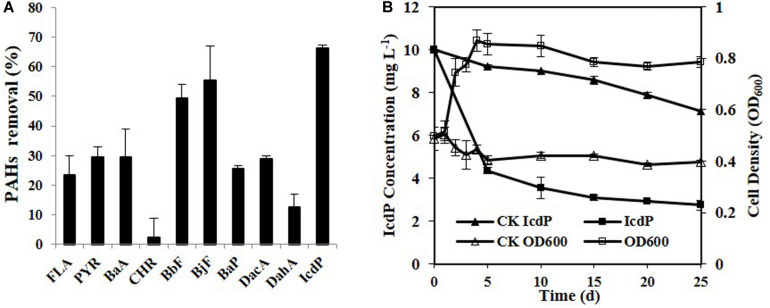
Degradation of various HMW PAHs (10 mg/L) by strain IcdP1 in MSM **(A)**, microbial growth, and IcdP degradation **(B)**; negative control was expressed as CK. Values are means ± SD (error bars) for three replicates.

Ten HMW PAHs were tested for their degradation by strain IcdP1. The results (Figure 1A) showed that strain IcdP1 exhibited a quite strong degrading ability to these substrates. Nine of the 10 HMW PAHs tested could be degraded, the only exception being CHR. In addition, the strain showed the highest degrading ability to IcdP, with a degradation percentage of 72.64 ± 2.2% after cultivation for 25 days. The removal rate of IcdP was more than 64.4 ± 5.1% even within 10 days (Figure 1B). The degradation percentages of four-ring HMW PAHs such as FLA, PYR, and BaA were 23 ± 6.5, 29 ± 3.5, and 29 ± 9.5%, respectively. The five-ring ones, including BbF, BjF, BaP, DacA, and DahA, were degraded by 49 ± 4.5, 55 ± 11.5, 25 ± 1, 29 ± 1, and 12 ± 4.5%, respectively (Figure 1A). Unlike other HMW PAH-degrading strains, IcdP1 preferred five- to six-ring HMW PAHs to four-ring ones and could grow on HMW PAHs as the sole carbon source.

### IcdP Degradation Through 7,8 and 1,2 Hydroxylation in Strain IcdP1

**FIGURE 2:**
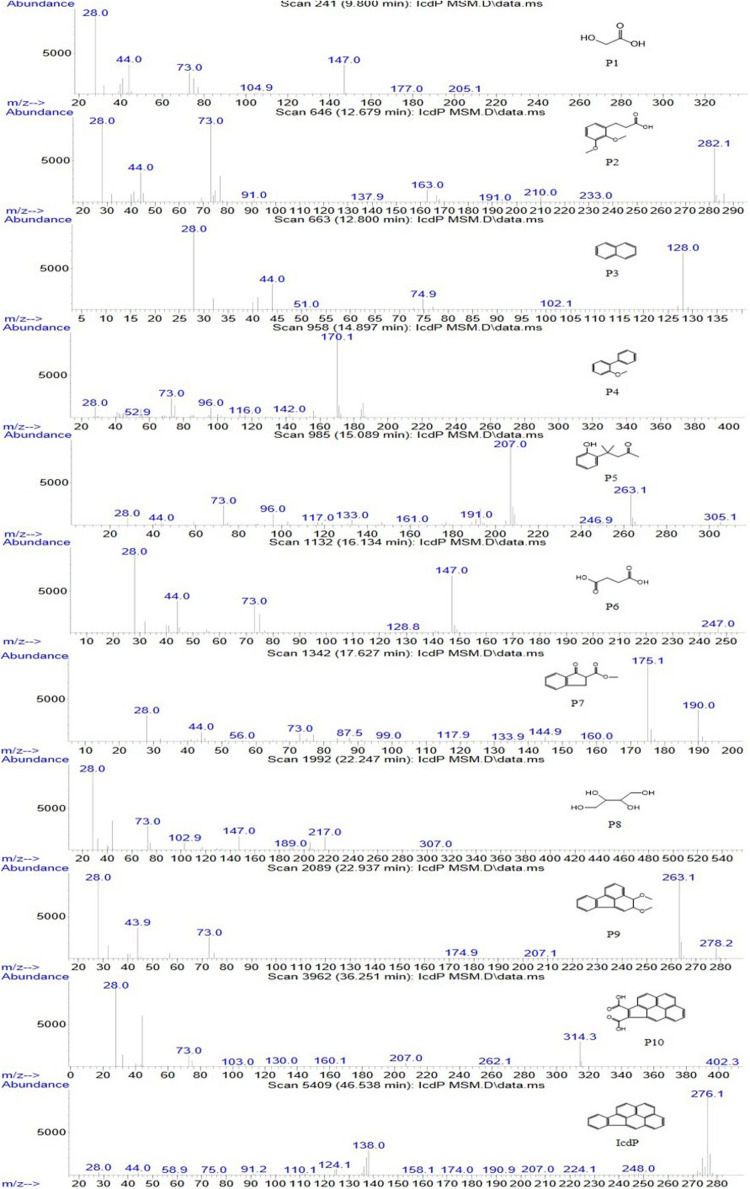
Mass spectra and structures of intermediates in the IcdP degradation pathway in strain IcdP1.

**FIGURE 3:**
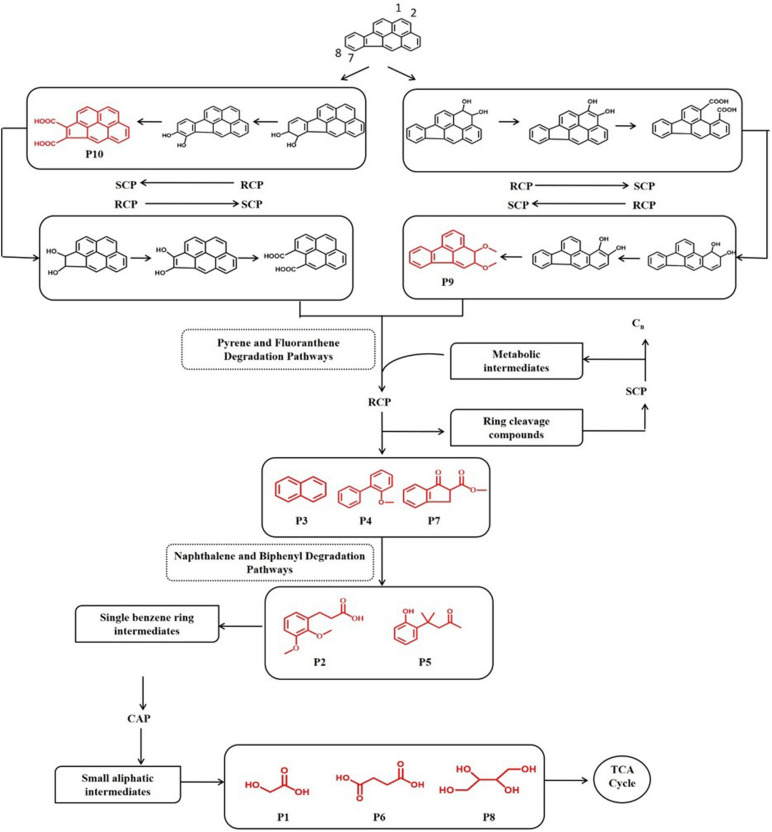
Proposed IcdP degradation pathways in strain IcdP1. Identified molecules through GC-MS are shown in red.

In order to investigate the degradation pathways of IcdP in strain IcdP1, the degradation intermediates were detected and identified by SPE-GC-MS. The results indicated that there were 10 degradation intermediates identified when strain IcdP1 grew and degraded IcdP as a sole carbon source (Table 2); their structures are shown in Figure 2. These intermediates included P10, cyclopenta[cd]pyrene-3,4-dicarboxylic acid; P9, 2,3-dimethoxy-2,3-dihydrofluoranthene; P4, 2-methoxy-1,1′-biphenyl, etc. (Table 2). Based on these detected metabolites, IcdP degradation pathways were proposed (Figure 3); IcdP was ring hydroxylation-initiated through at least two pathways. One was shown to occur *via* ring hydroxylation at the 7,8 positions of IcdP and side chain was removed, resulting in a five-ring product, cyclopenta[cd]pyrene-3,4-dicarboxylic acid (P10, Table 2 and Figure 3), a derivate of cyclopenta[cd]pyrene. The other occurred *via* 1,2 hydroxylation; in this pathway, IcdP was degraded to form a four-ring product, 2,3-dimethoxy-2,3-dihydrofluoranthene (P9, Table 2), a derivate of fluoranthene, through two rounds of ring hydroxylation and side chain removal. Products P10 and P9 were further metabolized through a pyrene-like or fluoranthene-like pathway and transformed into two-ring products, 2-methoxy-1,1′-biphenyl (P4, Table 2), a derivate of biphenyl, or naphthalene (P3, Table 2). Then, these two intermediates underwent biphenyl and naphthalene degradation pathways, which generated metabolites 3-(2,3-dimethoxyphenyl) propanoic acid (P2, Table 2) and 4-(2-hydroxypheny)-4-methylpentan-2-one (P5, Table 2) by RCP and SCP processes. These metabolites finally entered the CAP and transformed into low-molecular-weight fatty acids, such as 2-hydroxyacetic acid (P1, Table 2), succinic acid (P6, Table 2), and butane-1,2,3,4-tetraol (P8, Table 2), followed by several transformations into metabolites leading to the TCA cycle.

**TABLE 2:** Intermediates formed in the degradation of IcdP by strain IcdP1.

Rings	Product	*Rt* (min)	Ion fragments m/z	Identification or possible structure (TMS)^a^
0	P1	9.800	205, 177, 147, 73, 28	2-Hydroxyacetic acid
0	P6	16.134	247, 147, 73, 44, 28	Succinic acid
0	P8	22.247	307, 217, 189, 147, 103, 73, 44, 28	Butane-1,2,3,4-tetraol
1	P2	12.679	282, 233, 210, 163, 91, 73, 44, 28	3-(2,3-Dimethoxyphenyl)propanoic acid
1	P5	15.089	263, 207, 191, 96, 73, 28	4-(2-Hydroxyphenyl)-4-methylpentan-2-one
2	P7	17.627	190, 175, 176, 73, 44, 28	Methyl 1-oxo-2,3-dihydro-1*H*-indene-2-carboxylate
2	P3	12.800	128, 44, 28	Naphthalene
2	P4	14.897	185, 170, 142, 116, 96, 73, 28	2-Methoxy-1,1′-biphenyl
4	P9	22.937	264, 263, 207, 73, 44, 28	2,3-Dimethoxy-2,3-dihydrofluoranthene
5	P10	36.251	314, 73, 44, 28	Cyclopenta[cd]pyrene-3,4-dicarboxylic acid
6	IcdP^a^	46.538	276, 207, 138, 44, 28	Indeno[1,2,3-*cd*]pyrene

*^a^Indeno[1,2,3-cd]pyrene standard.*

### RHOs Initiated IcdP Degradation in Strain IcdP1

The degradation of six-ring HMW PAHs such as IcdP was more complex than LMW PAHs. Therefore, their degradation and the mechanism in bacteria were still rarely described. It was reported that the degradation of PAHs was commonly initiated by different RHOs (Luo et al., 2016). Multiple gene copies encoding Rieske non-heme RHOs (50 copies) and CYP450s (22 copies) were identified in the genome of strain IcdP1 (Qu et al., 2015). These RHOs and CYP450s in IcdP1 showed low homogeneity with each other or with known oxygenases from other strains, and this might contribute to its efficient degrading ability toward a lot of HWM PAHs.

**FIGURE 4:**
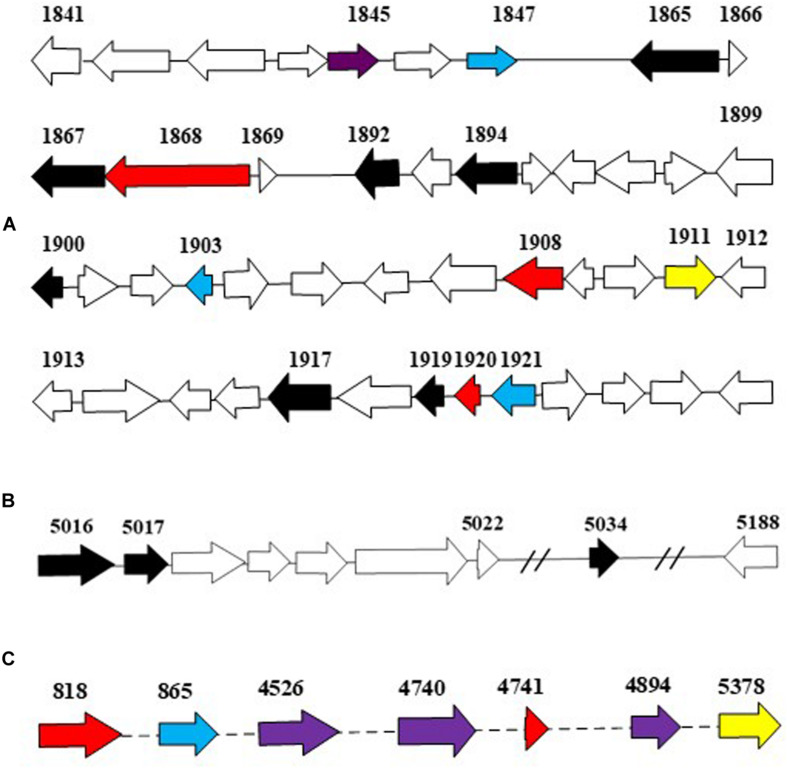
The putative dioxygenase in PAH degradation. Genes in blue were induced by PYR as the sole carbon source; genes in yellow were induced by BbF as the sole carbon source; genes in red were induced by IcdP as the sole carbon source; genes in purple were induced by both IcdP and PYR as the sole carbon source, respectively; genes in black were induced by IcdP, PYR, and BbF as the sole carbon source, respectively. **(A)**, the A region for PAH degradation; **(B)**, the B region for PAH degradation; **(C)**, the dispersed genes in genome that probably involved in PAH degradation.

The more abundant copies of RHOs than CYP450s inferred that the dioxygenation was the predominant pathway in the ring-hydroxylating degradation of HMW PAHs in strain IcdP1. Therefore, it was hypothesized that the expression of oxygenases that specifically catalyze the first cleavage of the ring of IcdP was merely or prominently induced by IcdP. In order to determine the enzymes involved in the first step of IcdP ring cleavages, the expression of all the RHOs and CYP450s was investigated using RT-PCR under the induction of IcdP, BbF, or PYR individually. The results indicated that the probable oxygenases involved in the first step of IcdP degradation were mainly distributed in PAH degradation regions A (1,977,937–2,019,582 bp) and B (5,386,471–5,435,990 bp) in the IcdP1 genome (also see Figure 4). Most of the oxygenases induced were RHOs and are mainly located in region A and partly in region B. Among them, the expression of the genes encoding RHOs 1908 and 1920 was only induced by IcdP by at least threefold higher than that of control. In addition, the genes for RHOs 818 and 4741, scattered in other region of the genome, were also only induced by IcdP (Figures 4A,C and Table 3). These results suggested that IcdP was degraded through a dioxygenation-initiated or monooxygenation-initiated metabolism pattern, and enzymes RHOs 1908, 1920, 818, and 4741 were possibly responsible for the first ring cleavage of IcdP through 1,2-dihydrodiol or 7,8-dihydrodiol and resulted in forming 2,3-dimethoxy-2,3-dihydrofluoranthene or cyclopenta[cd]pyrene-3,4-dicarboxylic acid. This may have resulted from the multiple gene copies encoding various initial oxygenase enzymes such as RHOs 1908, 1920, 818, and 4741. The genes encoding RHOs 1917, 1919, 1892, 1894, etc., were all highly expressed on BbF, PYR, or IcdP (Table 3). This result might suggest that they are involved in the downstream degradation of IcdP, such as the CAP. However, among the genes induced by BbF, PYR, or IcdP, the genes encoding RHOs 1919, 1917, and 1892 were highly induced by IcdP, but only slightly by PYR and BbF, inferring that these genes were necessary and efficient for the degradation of IcdP, especially the upstream degradation. In addition, there were some genes induced by both IcdP and PYR, such as the gene encoding RHO 4526; it was suggested that these genes were involved in the degradation steps between PYR and intermediates generated from IcdP and CAP. The results also showed that some genes such as the gene coding RHO genes 4849, 4740, and 1921 and CYP genes 865 and 1150, etc. were only induced by PYR (Table 3), and these genes might specifically catalyze the degradation of PYR, most probably the initial ring cleavage.

**TABLE 3:** Different expressed fold of RHOs and CYPs on IcdP, PYR, and BbF as the sole carbon source in strain IcdP1.

Gene	Function	Induction fold^a^					
		IcdP		Pyr		B(b)F	
		*recA*^b^	*rpoB*^b^	*recA*	*rpoB*	*recA*	*rpoB*
RHO818	2,3-Dihydroxybiphenyl 1,2-dioxygenase	1.85	4.73	–^c^	–	–	–
RHO4741	Aromatic-ring-hydroxylating dioxygenase, beta subunit	9.07	24.60	–	–	–	–
RHO1920	3-Phenylpropionate dioxygenase ferredoxin subunit	8.11	22.00	–	–	–	–
RHO4740	3-Phenylpropionate dioxygenase alpha subunit	–	–	1.71	2.28	–	–
RHO4894	Probable taurine catabolism dioxygenase	–	–	4.39	5.86	–	–
865	Putative cytochrome P450 hydroxylase	–	–	5.52	6.22	–	–
1150	Putative cytochrome P450 hydroxylase	–	–	2.16	2.37	–	–
RHO1921	2,3-Dihydroxy-2,3-dihydro-phenylpropionate dehydrogenase	–	–	3.02	2.96	–	–
RHO4526	2,3-Dihydroxybiphenyl 1,2-dioxygenase	2.16	1.97	2.32	2.29	–	–
RHO1892	2,3-Dihydroxybiphenyl 1,2-dioxygenase	4.16	11.39	2.05	3.52	2.72	3.63
RHO1894	Rieske (2Fe-2S) domain-containing protein	2.44	2.21	3.16	2.51	5.88	5.77
RHO1917	2,3-Dihydroxybiphenyl 1,2-dioxygenase	2.19	6.01	2.00	3.42	1.91	2.54
RHO1919	Biphenyl 2,3-dioxygenase	2.43	6.59	1.50	2.58	4.47	5.97

*^a^Only those increased for > 1.5-fold in both recA and rpoB as reference genes are identified as significant increase and listed here. ^b^recA or rpoB is used as reference gene for each RHO and CYP450. ^c^ –, indicated increase fold < 1.5 of recA or rpoB.*

### Sequence Alignment

**FIGURE 5:**
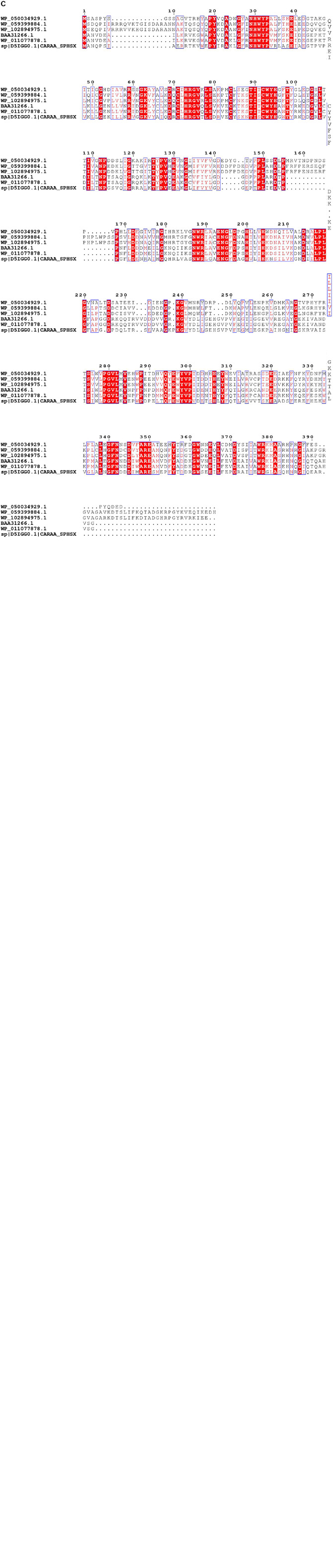
Sequence alignment of 1892 **(A)**, 1893 **(B)** and 1894 **(C)**, and their homologs from *Pseudomonas*, *Mycobacterium* sp., *Mycobacterium vanbaalenii*, *Rhodococcus* sp., *Rhodococcus jostii*, *Sphingomonas* sp., etc. Sequence alignment was performed using the ClustalW (v. 2.0) and ESPript programs. Conserved sequences are indicated by boxes, and similar sequences are indicated by colored background.

The RHOs induced by IcdP were analyzed by sequence alignment to further select the unique genes for ring hydroxylation initiation of IcdP. RHOs 1892–1894 were the subunits of a multi-subunit dioxygenase. Sequence alignment of RHOs 1892, 1893, and 1894, with their homologs from *Pseudomonas*, *Mycobacterium* sp., *Mycobacterium vanbaalenii*, *Rhodococcus* sp., *Rhodococcus jostii*, *Sphingomonas* sp., etc., revealed that they showed very low sequence similarity to their counterparts (Figure 5). RHO 1892, annotated as an aromatic ring-opening oxygenase LigA, exhibited highest identity (415) with biphenyl-2,3-diol 1,2-dioxygenase from *Pseudomonas putida* (Accession No. AAA25756.1). RHO 1893, annotated as benzoate 1,2-dioxygase or ferredoxin reductase, showed a quite low identity (39%) with a methane monooxygenase from *Pseudomonas* (Accession No. wp 059399896.1); RHO 1894, the third subunit of the multi-subunit dioxygenase, annotated as a Rieske (2Fe-2S) domain-containing protein, showed the highest identity of 47% with its counterpart from *P. putida* (Accession No. wp 096425533.1). RHOs 1917 (2,3-dihydroxybiphenyl-1,2 dioxygenase), 1919 (beta subunit of biphenyl dioxygenase), and 1920 (3-phenylpropionate dioxygenase ferredoxin subunit) showed maximum identity (44, 65, and 60%, respectively) with their homologs from *Mycobacterium vanbaalenin*. RHO 4740, annotated as beta subunit of an aromatic ring-hydroxylating dioxygenase, showed < 70% identity with its homolog from a *Rhodococcus* strain. Another RHO, 4741, a subunit of an aromatic ring-hydroxylating dioxygenase, showed < 53% identity with its homolog, which was annotated as an unknown protein in most bacterial species. Therefore, combining the above results of intermediates detected and transcription patterns of the genes induced by different substrates, it was considered that the degradation of IcdP in strain IcdP1 was initiated by ring hydroxylation at several positions, and the enzyme RHOs 1892–1894, 1917–1920, and 4740–4741 were involved in this initial ring hydroxylation.

## Discussion

PAHs are teratogenetic, carcinogenic, and mutagenic, and the more complex structures and metabolic pathways of HWM PAHs, especially IcdP, are even more toxic to human health and environment (Burchiel and Luster, 2001; Wong et al., 2018; Wirnkor et al., 2019). *R. aetherivorans* IcdP1 could efficiently degrade IcdP (> 64% within 10 days) and other HWM PAHs. IcdP1 could grow on several HMW PAHs as the sole carbon source, and preferred five- to six-ring HMW PAHs to four-ring ones. These properties are significantly different from other HMW PAH-degrading strains. To date, several bacteria, such as *M. vanbaalenii* PYR-1 mentioned above and *Sphingomonas yanoikuyae* JAR02 (Rentz et al., 2008), were described to have the ability to degrade FLA, PYR, BaA, BaP, etc., in a co-metabolism manner. For example, salicylic acid enhanced the degradation of BaP in *S. yanoikuyae* JAR02. However, few strains could degrade HMW PAHs as a sole carbon source, especially > 5-ring ones (Huang et al., 2016; Kong et al., 2018).

Multiple pathways of PYR, FLA, and BaP degradation have been reported in other strains such as *M. vanbaalenii* PYR-1 strain (Moody et al., 2004; Brezna et al., 2006; Kweon et al., 2007). It could oxidize PYR at the C-1,2 and C-4,5 positions by dioxygenase and monooxygenase, respectively. In addition, FLA at the C-1,2, C-2,3, and C-7,8 positions and BaP at the C-4,5, C-9,10, and C-11,12 positions. Based on the degradation intermediates such as cyclopenta[cd]pyrene-3,4-dicarboxylic acid (P10, Table 2 and Figure 3) and 2,3-dimethoxy-2,3-dihydrofluoranthene (P9, Table 2), the IcdP degradation was initiated by ring hydroxylation, at least, at the 1,2 and 7,8 positions. Through the pyrene-like or fluoranthene-like pathway, these metabolites are transformed into biphenyl or naphthalene (P3, Table 2) and then generated to low-molecular-weight fatty acids, such as 2-hydroxyacetic acid (P1, Table 2), succinic acid (P6, Table 2), and butane-1,2,3,4-tetraol (P8, Table 2). This phenomenon was consistent with the features that bacterial degradation of HMW PAHs presented multiple pathways, even for one chemical, although the substrate was usually converted into a limited number of central metabolic intermediates (Kim et al., 2007, 2008, Kweon et al., 2007, 2011). More abundant gene copies encoding Rieske non-heme RHOs (50 copies) than CYP450s (22 copies) inferred that the dioxygenation was the predominant pathway in the ring-hydroxylating degradation of HMW PAHs in strain IcdP1. Abundant gene copies encoding Rieske non-heme RHOs and CYP450s were a common phenomenon in bacteria; it is consistent with other strains such as *M. vanbaalenii*, etc., and it also inferred a common initial degrading mechanism and diverse degradation pathway of HMW PAHs in these species. The expression of genes encoding RHOs 1892–1894, 1917–1920, and 4740–4741 was induced strictly by IcdP, and the amino acid sequences of these proteins showed very low identities with their homologs. Therefore, RHOs 1892–1894, 1917–1920, and 4740–4741 responded to the initial ring cleavage of IcdP through 1,2-dihydrodiol or 7,8-dihydrodiol. The low identity of these RHOs with their homologs from other PAH-degrading bacteria suggested that strain Icdp1 possesses unique RHOs; apart from common initial degrading mechanisms for PAHs, new mechanisms also existed in strain IcdP1 for the degradation of IcdP as well as other HMW PAHs.

In summary, strain IcdP1 was isolated from a high-concentration IcdP content (>38 mg/kg) area; it showed high IcdP degradation ability and was even more efficient than lower PAHs (four rings). The detection of degradation intermediates such as cyclopenta[cd]pyrene-3,4-dicarboxylic acid and 2,3-dimethoxy-2,3-dihydrofluoranthene and the high expression of genes encoding RHOs on IcdP as substrate inferred that IcdP degradation was initiated by ring hydroxylation, at least, at the 1,2 and 7,8 positions. These properties were quite different from other strains or had not been reported in other researches.

The ability of strain IcdP1 to degrade a wide range of HMW PAHs (four to six rings, especially IcdP) is valuable not only in the study on the degradation and molecular mechanism of HMW PAHs but also in the bioremediation of environments polluted by PAHs.

The metabolic and transcriptional profiles in HWM PAH pathways would contribute to the understanding of the molecular mechanism of initial biodegradation of IcdP in *R. aetherivorans* and other strains.

## Conclusion

In this study, IcdP degradation pathways were determined to be ring hydroxylation-initiated through the 7,8 and 1,2 positions in an IcdP-degrading strain, *R. aetherivorans* IcdP1. The first step of IcdP ring cleavage was catalyzed mainly by aromatic ring-hydroxylating dioxygenases (RHOs). The expression of RHOs 1908, 1920, 818, and 4741 was only highly induced by IcdP, and these RHOs were the key initial aromatic RHOs responsible for the first ring cleavage of the IcdP through 1,2-dihydrodiol or 7,8-dihydrodiol. The studies would contribute to our understanding of the molecular mechanism of initial deoxygenation of IcdP degradation and provide the clues for the usage of strain IcdP1 in the bioremediation of PHA-contaminated environments.

## Data Availability Statement

The original contributions presented in the study are included in the manuscript/supplementary material, further inquiries can be directed to the corresponding author/s.

## Author Contributions

L-LM and JQ conducted the experiments and analyzed the samples and data. L-LM wrote the manuscript with significant assistance and comments from all the other authors. Z-PL supervised, designed, and revised the manuscript. All authors approved the final version of the manuscript.

## Conflict of Interest

The authors declare that the research was conducted in the absence of any commercial or financial relationships that could be construed as a potential conflict of interest.

## Abbreviations

BaA: Benz[a]anthracene
BaP: Benzo[a]pyrene
BbF: Benzo[b]fluoranthene
BjF: Benzo[j]fluoranthene
CHR: Chrysene
CYP450s: Cytochrome P450 monooxygenases
DacA: Dibenz[a,c]anthracene
FLA: Fluranthene
HMW PAHs: High-molecular-weight PAHs
IcdP: Indeno[1,2,3-cd]pyrene
LMW PAHs: Low-molecular-weight PAHs
PAHs: Polycyclic aromatic hydrocarbons
PYR: Pyrene
RHOs: Aromatic ring-hydroxylating oxygenases.
